# The anti-apoptotic, antioxidant and anti-inflammatory effects of curcumin on acrylamide-induced neurotoxicity in rats

**DOI:** 10.1186/s40360-020-00440-3

**Published:** 2020-08-18

**Authors:** Jie Guo, Xiaolu Cao, Xianmin Hu, Shulan Li, Jun Wang

**Affiliations:** 1 Hubei Province Key Laboratory of Occupational Hazard Identification and Control, Wuhan University of Science and Technology, Wuhan, 430065 China; 2 Department of Pharmacy, New Medicine Innovation and Development Institute, College of Medicine, Wuhan University of Science and Technology, Wuhan, 430065 China

**Keywords:** Acrylamide, Curcumin, Apoptosis, Antioxidant, Inflammation, Telomerase reverse transcriptase

## Abstract

**Background:**

Acrylamide (ACR) formed during heating of tobacco and carbohydrate-rich food as well as widely applied in industries has been known as a well-established neurotoxic pollutant. Although the precise mechanism is unclear, enhanced apoptosis, oxidative stress and inflammation have been demonstrated to contribute to the ACR-induced neurotoxicity. In this study, we assessed the possible anti-apoptotic, antioxidant and anti-inflammatory effects of curcumin, the most active component in a popular spice known as turmeric, on the neurotoxicity caused by ACR in rats.

**Methods:**

Curcumin at the dose of 50 and 100 mg/kg was orally given to ACR- intoxicated Sprague-Dawley rats exposed by ACR at 40 mg/kg for 4 weeks. All rats were subjected to behavioral analysis. The HE staining and terminal deoxynucleotidyl transferase mediated dUTP nick end labelling (TUNEL) staining were used to detect histopathological changes and apoptotic cells, respectively. The mRNA and protein expressions of apoptosis-related molecule telomerase reverse transcriptase (TERT) were detected using real-time PCR and immunohistochemistry, respectively. The contents of malondialdehyde (MDA) and glutathione (GSH) as well as the activities of superoxide dismutase (SOD) and glutathione peroxidase (GSH-Px) were measured as the indicators for evaluating the level of oxidative stress in brain. The levels of pro-inflammatory cytokinestumor necrosis factor-α (TNF-α) and interleukin-1β (IL-1β) in the cerebral homogenates were detected using ELISA assay.

**Results:**

ACR-induced weigh loss, deficits in motor function as well as pathological alterations in brains were significantly improved in rats administrated with 50 and 100 mg/kg curcumin. TUNEL-positive apoptotic cells in curcumin-treated ACR intoxicated brains were less than those in the ACR model group. Curcumin administration especially at the dose of 100 mg/kg upregulated the TERT mRNA expression and enhanced the number of TERT-positive cells in ACR-intoxicated cortex tissues. Moreover, curcumin treatment reduced the concentrations of TNF-α, IL-1β and MDA, while increased the GSH contents as well as the SOD and GSH-Px activities in the cerebral homogenates, in comparison to ACR control group.

**Conclusions:**

These data suggested the anti-apoptotic, antioxidant and anti-inflammatory effects of curcumin on ACR-induced neurotoxicity in rats. Maintaining TERT-related anti-apoptotic function might be one mechanism underlying the protective effect of curcumin on ACR-intoxicated brains.

## Background

As a chemical formed during the high-temperature processing of tobacco and carbohydrate-rich foods, acrylamide (ACR) is well recognized as a human neurotoxin which has posed significant public health concerns due to its daily intake [1–3]. Moreover, ACR is widely employed in various chemical and industrial processes as a component to produce polymers used in gel chromatography, dye synthesis, production of paper, cosmetics and waste water management, *etc* [4, 5]. The work-related ACR exposure has been demonstrated to bring on neurotoxicity in occupationally exposed population, which is manifested as ataxia, skeletal muscle weakness, gait abnormalities, skin abnormalities, as well as numbness of hands and feet [4].

The exposure to monomeric form of ACR results in multiple pathological changes in central and peripheral nervous system. Among them, ACR-induced apoptosis that subsequently leads to the death and loss of neurons has been accepted as a fundamental and predominant mechanism of neurotoxicity in ACR-exposed humans and animals [6–8]. Telomerase reverse transcriptase (TERT) is one of catalytic units of telomerase, importantly, acts as rate-limiting determinant and the most important regulator of telomerase activity [9, 10]. Telomerase is required to synthesize the telomeric DNA strand thus maintain the length of telomeres, the latter of which is a DNA-protein complex located at chromosome ends and has an ability of protecting against genome instability [9]. So far, the anti-apoptotic effect of TERT has been revealed in neuronal cells influenced by various risk factors such as oxidative stress, DNA damage and ischemia [9, 10]. In line with these findings, our previous study [5] has demonstrated that TERT-related anti-apoptotic function was significantly down-regulated in rats with ACR-induced neurobehavioral deficits. The mRNA and protein expression of TERT in the rat cerebral cortex was reduced by ACR treatment [5]. As the critical events in chemical-induced neurodegeneration, oxidative stress and enhanced lipid peroxidation are induced by exposure to ACR, which are also important mechanisms underlying ACR-induced neurotoxicity [11, 12]. During ACR metabolism in the body, excessive levels of reactive oxygen species (ROS) are certainly produced. Moreover, ACR may have deleterious effects on antioxidant enzymes such as superoxide dismutase (SOD) and glutathione peroxidase (GSH-Px) thus decrease the antioxidant defence in the brains [11, 12]. Furthermore, many evidences [12, 13] have shown the production of inflammatory cytokines such as tumor necrosis factor-α (TNF-α) and interleukin-1β (IL-1β) was enhanced after ACR intoxication.

Accordingly, in recent years, some agents with anti-apoptosis, antioxidant and anti-inflammatory properties have been expected to attenuate ACR-induced neurotoxicity [3, 8, 11–14]. As the most active constituent in turmeric, a common spice, with a strong safety record, curcumin has been considered to be a potential natural neuroprotective agent under limelight [15–18]. Based on its known antioxidant, anti-inflammatory and anti-apoptosis activities, curcumin has been shown to protect the neurons against cerebral ischemia-reperfusion injury [15, 16], dysfunction linked with Parkinson’s disease mediated by Bisphenol-A [19], sleep-deprivation induced memory impairments [20], and depression [21], etc. However, there is limited evidence in the possible ameliorative effect of curcumin against ACR-induced neurotoxicity. Prasad and Muralidhara [22] have demonstrated the neuroprotective effect of curcumin in an ACR model of neurotoxicity in an insect species, *Drosophila melanogaster*. A recently published study [23] reported that curcumin would exert a protective effect against ACR-induced spatial memory impairment in rats. However, the anti-apoptotic, antioxidant and anti-inflammatory activities of curcumin have not been well evaluated in ACR-induced neurotoxicity. In the present study, we identified whether curcumin could exert protective effects against neuron apoptosis, oxidative stress and inflammatory response caused by ACR exposure in rats.

## Methods

### Chemicals

ACR and curcumin were purchased from Amresco Co. (Solon, OH, USA) and Sigma chemicals Co.(St. Louis, MO, USA), respectively.

### Experimental design

Male Sprague-Dawley rats, weighing 200–220 g, were obtained from Hubei Experimental Animal Research Center (Hubei, China). Rats were housed in standard translucent cages (5 animals/cage) under controlled standard conditions (23 ± 2 °C, 55 ± 5% relative humidity, 12 h light/dark cycle) with restricted access to standard rat chow and free access to tap water. After acclimation for 1-week, healthy animals were randomly assigned into 4 groups (10 rats per group): normal control group; ACR-intoxicated control group; low-dose (50 mg/kg) curcumin treatment group and high-dose (100 mg/kg) curcumin treatment group. A dose of 40 mg/kg ACR (dissolved in normal saline) was intraperitoneally injected every other day for 4 weeks in all animals except the normal control group. The normal rats received saline as control. Meanwhile, rats in the curcumin treatment groups were daily administered with curcumin at the corresponding oral administration dose for 4 weeks. The doses of ACR and curcumin were chosen based on the previous study [5] and preliminary experiments. The normal and ACR-intoxicated control animals were orally administered with the same volume of distilled water. Body weight and behavioral alterations were monitored once a week. At 24 h after the last administration, all animals were euthanized by CO_2_ asphyxiation, brain tissues were quickly collected.

### Behavioral tests

All rats were subjected to behavioral analysis to assess their motor functions.

In the hind limb splay examination [3, 5], the hind paws of rats were inked, then the rats were placed in a horizontal position of 30 cm high and dropped onto a white paper. The distance between the center points of right and left heels were recorded as the landing foot spread distance.

In the movement initiation test [5, 24], the rat was held by its hind limbs and its torso, one forelimb was lifted above a table in order that the body weight was supported by the other forelimb alone. Then, rat was allowed to initiate stepping movements for one forelimb, and then the other. The averaged time period to initiate one step was recorded as the response latency for each forelimb.

In the gait score test [3, 5], animals were placed on the table and were observed for 3 min. Gait was scored as follow: 1: normal gait; 2, slightly abnormal gait characterized by slight ataxia, weakness and foot splay; 3, moderately abnormal gait characterized by obvious ataxia and foot splay with limb spread during ambulation; 4, severely abnormal gait characterized by a combination of all the above symptoms, dragging hind limbs and inability to support body weight.

### Histopathological analysis

The collected brain tissues were fixed with 10% neutral-buffered formalin followed by dehydrating and paraffin-embedding. Then, embedded brain sections (5-μm thickness) were stained with hematoxylin and eosin (HE) for histopathological observation. The histopathological changes in cerebral cortex, hippocampal CA1, CA3, and dentate gyrus regions were analyzed.

### TUNEL assay

The apoptotic neurons in the brain sections were detected using the terminal deoxynucleotidyl transferase mediated dUTP nick end labelling (TUNEL) assay. After deparaffinization and rehydration, the brain sections were permeabilized with proteinase K solution, then exposed to the mixture of biotinylated nucleotide dUTP and recombinant terminal deoxynucleotidyl transferase (TdT) following the instruction manual of TUNEL Apoptosis Assay Kit (Servicebio, Wuhan, China). Staining with 4,6-diamino-2-phenyl indole (DAPI) (Sigma, St. Louis, USA) was performed to visualize nuclei. Images were obtained under a fluorescent microscope (Olympus, Center Valley, USA).

### Real-time PCR

Total RNA of brain cerebral cortex tissues was isolated using TRIzol reagent (Invitrogen, Carlsbad, CA, USA). The expression levels of TERT mRNA were measured by real-time PCR using all-in-OneTM qPCR master mix AOPR-1200 (GeneCopoeia, Rockville, MD, USA). The sequences of primer sets for TERT were 5′-TGTTCCTGTTCTGGCTAATGG- 3′(forward) and 5′-CCTCTTGTGACAGTTCCCGT-3′ (reverse). β-actin gene was applied as a reference.

### Immunohistochemistry

Paraffin-embedded brain sections of 5-μm thickness were incubated with a rabbit anti-TERT antibody (Servicebio, Wuhan, China), then a biotinylated goat anti-rabbit secondary antibody (Servicebio, Wuhan, China). Immune complexes were visualized by incubation with 3,3′-diaminobenzidine tetrachloride (DAB) and hematoxylin.

### Measurement of parameters related to oxidative stress in cerebral homogenates

The brain tissue were homogenized with 9 times the volume of PBS on ice and then centrifuged to prepare homogenates. The contents of malondialdehyde (MDA) and glutathione (GSH) as well as the activities of SOD and GSH-Px in the cerebral homogenates were measured following the respective manufacturer’s protocols (Nanjing Jiancheng Bio-Engineering Co., Ltd., Nanjing, China). Protein contents in the cerebral homogenates were determined using the bicinchoninic acid assay kit (Nanjing Jiancheng Bio-Engineering Co., Ltd., Nanjing, China).

### Measurement of IL-1β and TNF-ɑ levels in cerebral homogenates

The concentrations of IL-1β and TNF-ɑ in cerebral homogenates were determined using using ELISA kits according to the manufacturer’s instructions (IL-1β: PeproTech Inc., NJ, USA; TNF-ɑ: R&D Systems, Minneapolis, MN, USA).

### Statistical analysis

All experiments were conducted with two technical replicates. Data were expressed as the mean ± SD, and analyzed using one-way analysis of variance (ANOVA) with post hoc Tukey test by SPSS 22.0 software. *P* < 0.05 or *P* < 0.01 was considered statistically significant.

## Results

### Effect of curcumin on ACR-induced body weight and neurobehavioral changes

**Fig. 1:**
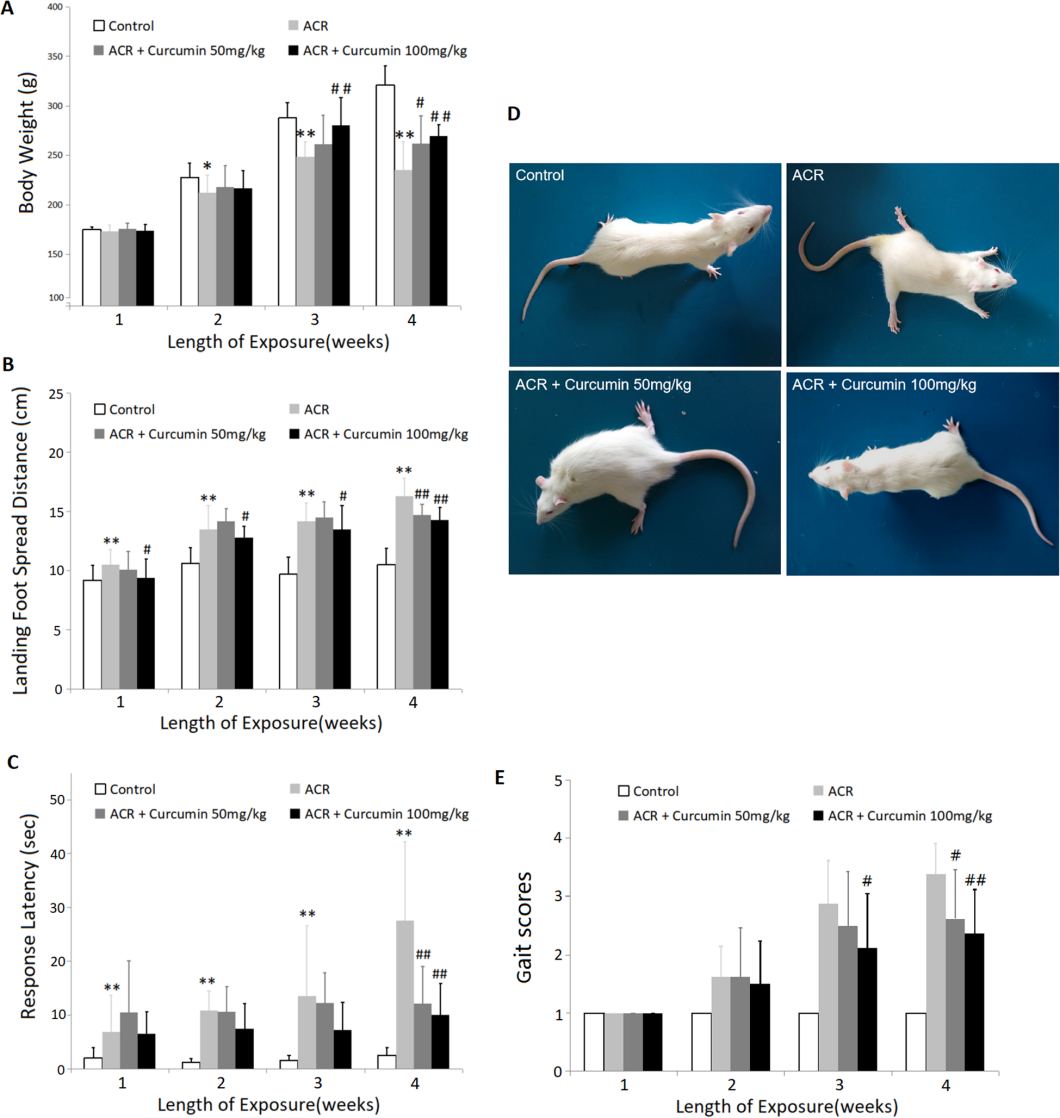
Effect of curcumin on the body weights (**a**), landing foot spread distance (**b**), movement initiation test (**c**) and gait (**d** and **e**) in ACR-treated rats. Data are means±SD of 10 animals in each group. *P < 0.05, **P < 0.01 compared to the corresponding control rats. ^#^ *P* < 0.05, ^##^ *P* < 0.01; compared with the corresponding ACR group

As shown in Fig. 1a, the animals in the ACR group began to show slow growth compared to the normal control group since 2 weeks of exposure (*P* < 0.05). At the end of the 4-week exposure period, the average body weight of ACR intoxicated rats was 73.4% of that of normal rats (*P* < 0.01). However, curcumin administration protected the rats from ACR-induced weigh loss. Compared with the ACR model group, curcumin at the dose of 50 mg/kg caused a significant weight gain at 4th week (*P* < 0.05). And the body weight of rats administrated with 100 mg/kg curcumin increased by 12.5 and 14.6% at 3rd and 4th week, respectively (*P* < 0.01).

Landing foot spread distance was enlarged rapidly from the first week of ACR exposure (Fig. 1b), and significant differences were found between the ACR intoxicated group and the normal control group throughout the exposure period (*P* < 0.01). Similarly, ACR intoxicated rats developed a progressive impairment of forelimb movement initiation (*P* < 0.01) (Fig. 1c) and significant gait abnormalities (Fig. 1d and e) including obvious ataxia and foot splay, twisting of hind limbs and inability to support body weight. Curcumin intervention in ACR intoxicated rats markedly improved these neurobehavioral changes in a dose-dependent manner (*P* < 0.05; *P* < 0.01).

### Effect of curcumin on ACR-induced histopathological alterations in rat brains

**Fig. 2:**
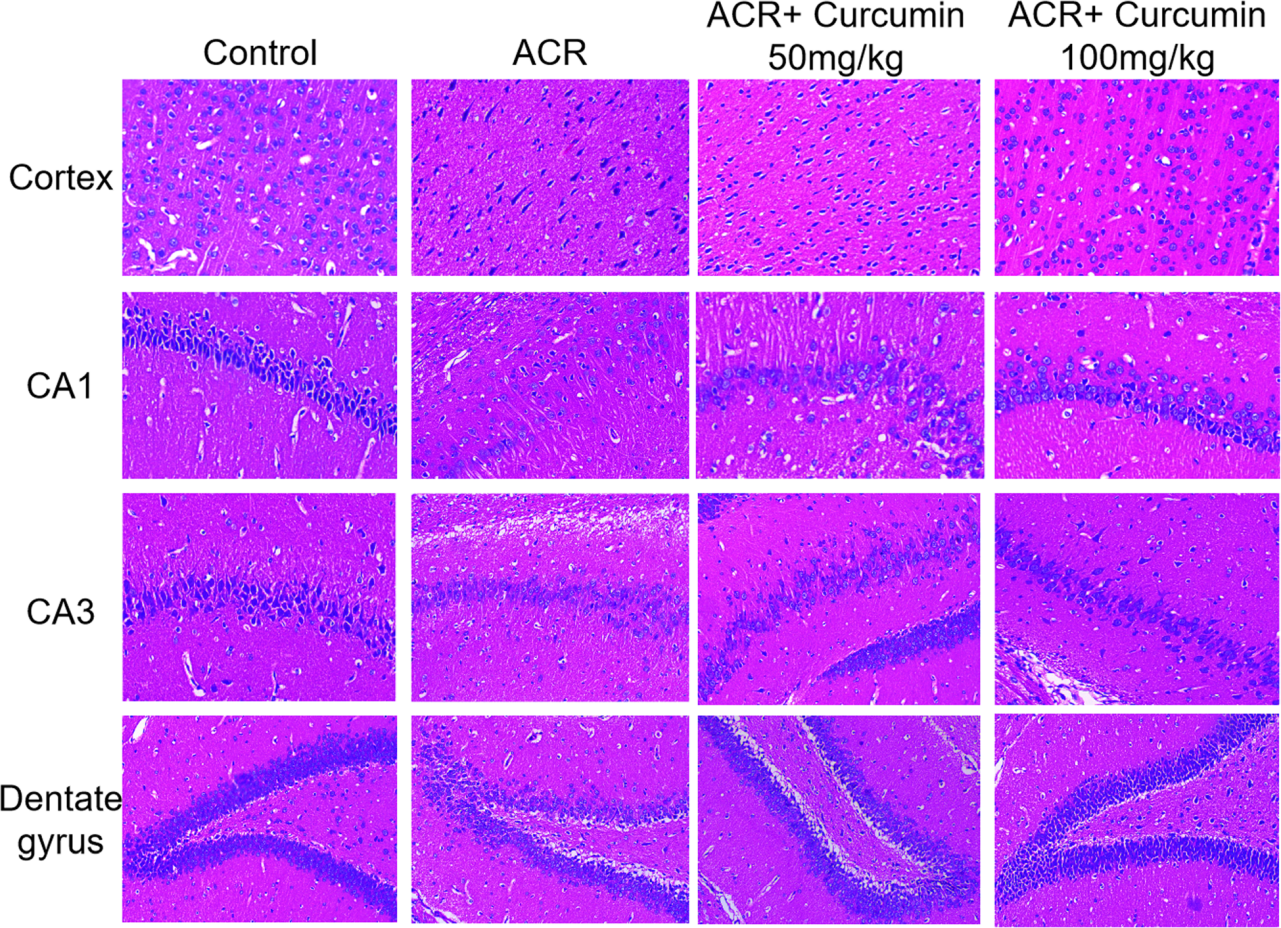
Effect of curcumin on the histopathological changes in cortex, CA1, CA3, dentate gyrus of ACR-treated rat brains. (H&E staining 200×)

The neuronal morphological characteristic in the cerebral cortex and hippocampus was identified using H&E staining. As showing in Fig. 2, severe neuronal loss, condensed and fragmented nuclei were found in the cortex and hippocampus of ACR intoxicated rats. Compared with the ACR model group, there was more nerve cells and less pathological alterations in the brain of rats administrated with curcumin.

### Protective effect of curcumin on ACR-induced neuron apoptosis

**Fig. 3:**
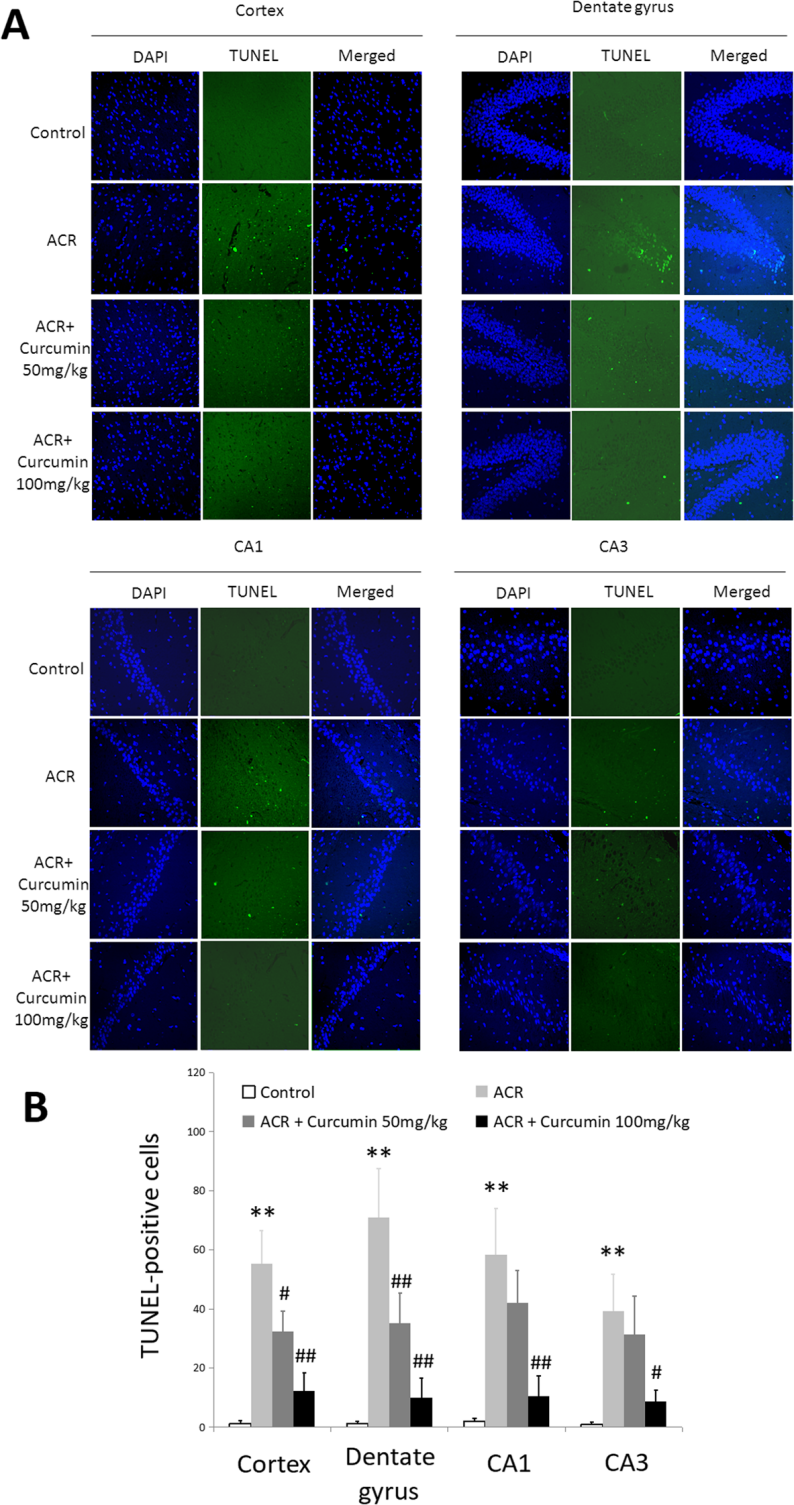
Effect of curcumin on the neuron apoptosis in ACR-treated rat brains. (TUNEL staining 400×). **a** Representative images. **b** Quantitative assessment of neuronal density of TUNEL-positive cells (number of cells/mm^2^). Data are means±SD of 10 animals in each group. **P < 0.01 compared to the corresponding control rats. ^#^ *P* < 0.05, ^##^ *P* < 0.01; compared with the corresponding ACR group

As showing in Fig. 3, immunofluorescent staining showed that the number of TUNEL-positive apoptotic nerve cells was significantly increased in the cortex and hippocampus of ACR intoxicated rats. However, curcumin administration could effectively reduce the number of apoptotic cells (*P* < 0.05; *P* < 0.01), suggesting its anti-apoptotic activity in ACR-damaged neurons. TUNEL-positive cells in curcumin-treated ACR intoxicated brains had decreased to approximately 13.8–22.1% of those in the ACR model group.

### Effect of curcumin on ACR-inhibited TERT expression

**Fig. 4:**
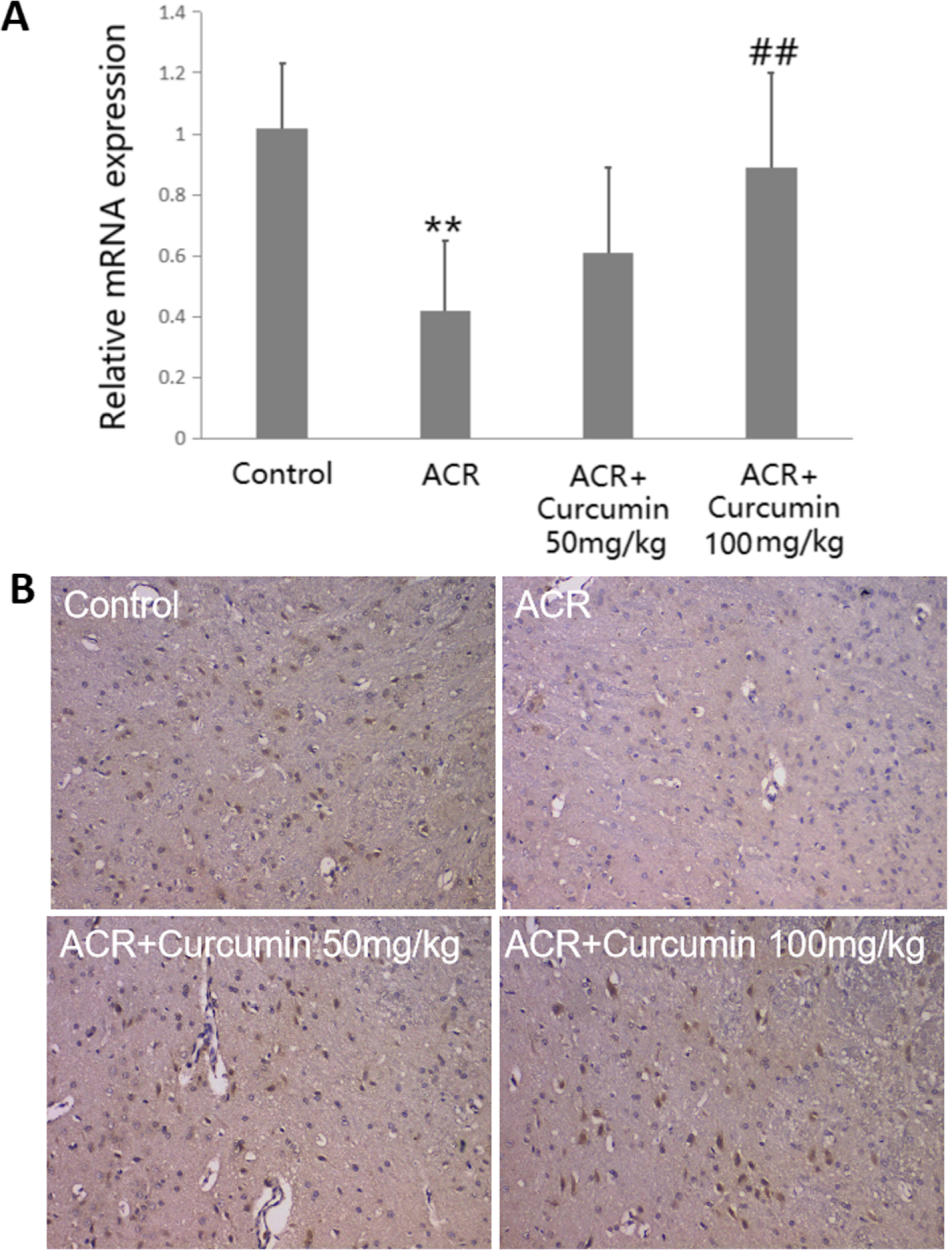
Effect of curcumin on the expression of TERT in the cortex tissues of ACR-treated rats. **a** The mRNA expression was measured with Real-time PCR. **b** Immunohistochemical staining for the protein expression of TERT. Data are means ± SD of 10 animals in each group. **P < 0.01 compared to the corresponding control rats. ^##^*P* < 0.01; compared with the corresponding ACR group

Our previous study [5] suggested that TERT, an emerging anti-apoptotic molecule mainly expressed in cortical neurons, was down-regulated in the cerebral cortex of ACR treated rats. In order to identify whether curcumin has regulative effect on ACR-inhibited TERT expression, the mRNA and protein expressions of TERT were detected using real-time PCR and immunohistochemistry, respectively. As shown in Fig. 4, curcumin treatment especially at the dose of 100 mg/kg increased TERT mRNA expression level (*P* < 0.01), and enhanced the number of TERT-positive cells in ACR-intoxicated cortex tissues, suggesting curcumin might exert anti-apoptotic activity in ACR-induced neurotoxicity partly through maintaining TERT-related anti-apoptotic function.

### Effect of curcumin on oxidative stress caused by ACR

To explored the possible anti-oxidant effect of curcumin on ACR-induced neurotoxicity in rats, the contents of MDA, GSH and the activities of SOD, GSH-Px in the cerebral homogenates were quantified as measures of the level of oxidative stress in the brain. As shown in Table 1, the content of MDA was markedly increased, while the GSH level, the activities of SOD and GSH-PX were markedly decreased in cerebral homogenates of ACR-treated rats in comparison to the normal control group (*P* < 0.01), suggesting ACR-induced oxidative stress in the brain. As expected, these alterations induced by ACR were significantly ameliorated by curcumin treatment in a dose-dependent manner (*P* < 0.05; *P* < 0.01), suggesting that the anti-oxidative activity of curcumin might, at least partly, be responsible for its neuroprotective effect in ACR intoxicated rats.

**Table 1:** Effect of curcumin on the levels of MDA, GSH, SOD and GSH-Px in cerebral homogenates prepared from ACR intoxicated rats (*n* = 10, mean ± SD)

Groups	MDA (nmol/mg prot)	GSH (mg/g prot)	SOD (U/mg prot)	GSH-Px (U/mg prot)
Normal	0.425 ± 0.141	4.41 ± 0.58	60.21 ± 5.38	14.81 ± 1.95
ACR	1.133 ± 0.352 **	2.30 ± 0.47**	52.72 ± 6.94 **	10.36 ± 1.84 **
ACR + curcumin 50 mg/kg	0.918 ± 0.322	2.77 ± 0.46 ^#^	53.89 ± 8.02	12.58 ± 1.96 ^#^
ACR + curcumin 100 mg/kg	0.854 ± 0.216 ^#^	2.92 ± 0.59 ^#^	59.16 ± 6.46 ^#^	13.15 ± 1.87 ^##^

**P < 0.01 compared to the corresponding control rats. ^#^ *P* < 0.05, ^##^ *P* < 0.01; compared with the corresponding ACR group

### Effect of curcumin on cerebral contents of IL-1β and TNF-ɑ in ACR intoxicated rats

**Fig. 5:**
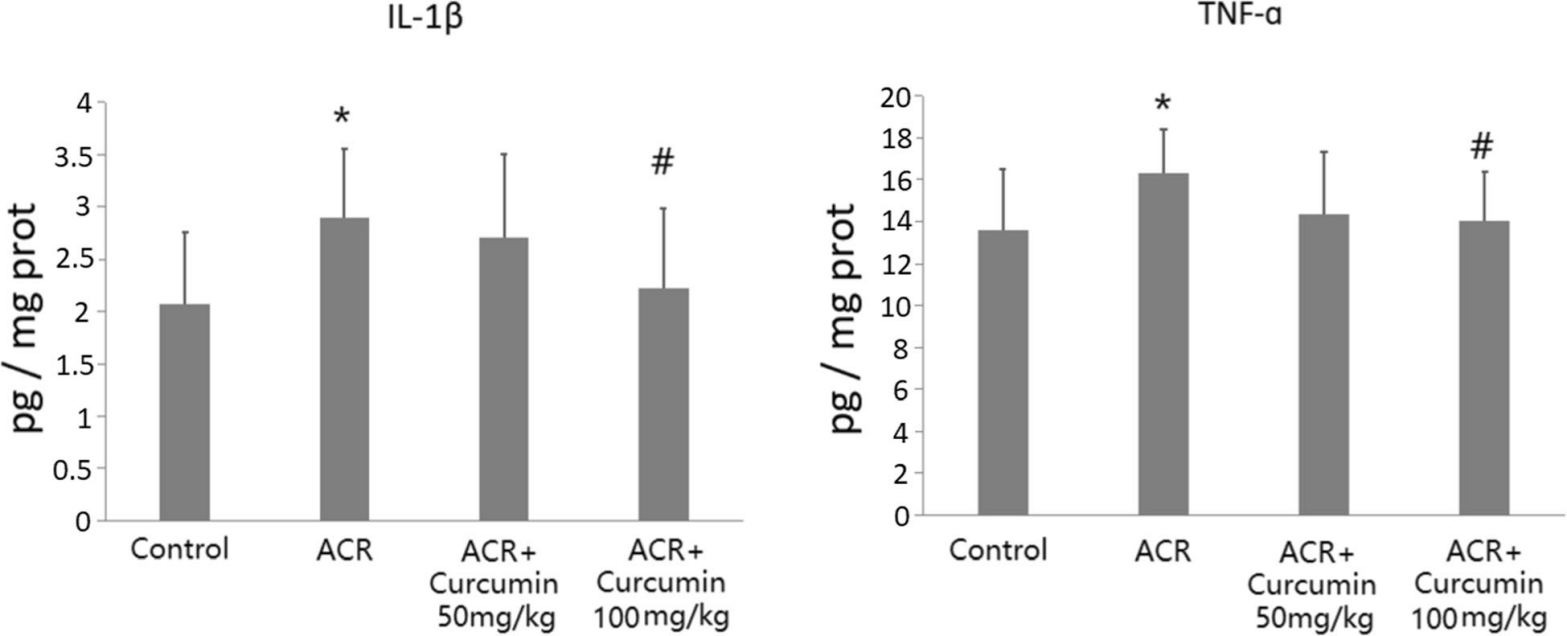
Effect of curcumin on cerebral contents of IL-1β and TNF-ɑ in ACR intoxicated rats. Data are means ± SD of 10 animals in each group. *P < 0.05 compared to the corresponding control rats. ^#^ *P* < 0.05; compared with the corresponding ACR group

To explore the possible anti-inflammatory activity involved in curcumin mediated neuroprotection in ACR intoxicated rats, the levels of pro-inflammatory cytokines IL-1β and TNF-ɑ were detected in the cerebral homogenates. Our results show that, although ACR exposure moderately stimulated the production of pro-inflammatory cytokines in brain (*P* < 0.05), curcumin at the dose of 100 mg/kg significantly decreased the levels of IL-1β and TNF-ɑ by 22.8 and 14.1%, respectively (*P* < 0.05) (Fig. 5), when compared with the ACR group.

## Discussion

Curcumin, with its neuroprotective effects and hardly existing toxicity, have become an attractive alternative treatment tool for various neurological disorders [15–20]. After systemic administration, curcumin can across the blood–brain barrier, and exert its therapeutic efficacy in the brain [25]. In the present study, we demonstrated the anti-apoptotic, antioxidant and anti-inflammatory effects of curcumin on ACR-induced neurotoxicity in rats, suggesting the use of curcumin to prevent or delay neurological damages induced by ACR exposure. In line with the evidences from humans and animals [4, 5, 8, 11–14], our study showed that the 4-week exposure of rats to ACR at the dose of 40 mg/kg caused a significant body weight loss, progressive deficits in motor function and adverse pathological outcome in the cortex and hippocampus of rats. Importantly, the present data revealed that curcumin administration could efficiently rescue ACR-induced weight loss and neurobehavioral deficits, relieve the neuropathological damages in brain.

As an important event of neuronal cell number control, apoptosis that is an inappropriate activation of the neuronal cell-suicide program has been well-accepted as a fundamental component in the development of various brain diseases [26]. In particular, in view of the very limited regenerative capacity of the central nervous system tissue, it is vitally important to prevent against neuronal cell apoptosis, and then limit the brain damage caused by neuronal death [26]. So far, apoptosis has become a prime therapeutic target in the development of neuroprotective agents. Treatment preventing the neuronal cell apoptosis can maintain the cell numbers, reduce the severity and progression of brain diseases. In the present study, the anti-apoptotic potential of curcumin in ACR-intoxicated brains which was manifested by the significant decreased TUNEL-positive apoptotic nerve cells in the cortex and hippocampus might be an important mechanism underlying its neuroprotective effect against exposure to ACR.

A variety of small molecules can act on crucial checkpoints of apoptosis [26]. In recent years, the role of TERT in apoptosis has attracted considerable interest as an emerging anti-apoptotic molecule involved in compensatory neuroprotective mechanism against neuronal cell death [9, 10]. ACR intoxication significantly reduced the expression of TERT in the brain, suggesting the TERT-related anti-apoptotic function participated in the ACR neurotoxicity [5]. Interestingly, some new evidences showing that curcumin up-regulates function of TERT have emerged [27, 28]. Curcumin extracted with ethyl acetate concentration-dependently up-regulated the TERT mRNA expression in rat clone-9 hepatocytes [27]. Pirmoradi et al. [28] reported that the TERT expression of rat adipose tissue-derived stem cells was significantly increased in the presence of curcumin at concentrations of 1 and 5 μM. In line with these in vitro studies [27, 28], we showed the curcumin-induced in vivo up-regulation of TERT at the levels of gene and protein, which might be one mechanism underlying the anti-apoptotic activity of curcumin in ACR-intoxicated brains.

In addition, curcumin is well known for its classic and strong anti-oxidative and anti-inflammatory activities [29]. ACR exposure has been demonstrated to result in a disturbance in the balance between the free radical formation and elimination, the latter of which is mediated by antioxidant systems [11, 12]. The phenolic structure in curcumin confers electron-capturing properties, which destabilize ROS, explaining the well-accepted antioxidant effects [30]. However, being similar to other antioxidants including vitamin E, vitamin C, and carotenoids, curcumin has been found to show double-edged roles in the level of intracellular ROS, which appeared to be highly dependent on the cell type [30–32]. Curcumin has been reported to elevate ROS levels in multiple cancer cells [30–32]. In this study, in line with the well-accepted anti-oxidative activity of curcumin in normal and non-malignant cells [29–32], 4-week exposure of rats to 40 mg/kg ACR markedly enhanced the level of MDA (an essential biomarker of oxidative stress and lipid peroxidation), decreased the content of GSH (a biologically important intracellular thiol acting as a free radical scavenger) and the activities of SOD and GSH-Px (two important antioxidant enzymes) in the brain tissues. But curcumin alleviated the augmented production of MDA and the reduction of antioxidant capacity induced by ACR, thus might play a role in the detoxification of reactive oxygen species generated by ACR. Moreover, neuroinflammation has been demonstrated in various pathologies of the brain including ACR-induced neurotoxicity [33]. The 4-week exposure to ACR induced inflammatory responses in the brain tissues, evident by upregulated levels of IL-1β and TNF-ɑ, two potent pro-inflammatory cytokines acting as master regulators of neuroinfammation in the central nerve system. While curcumin could improve the ACR-induced neuroinflammation, which was in accord with its proven anti-inflammatory property.

## Conclusions

In summary, this study convinced the anti-apoptotic, antioxidant and anti-inflammatory effects of curcumin on ACR-induced neurotoxicity in rats. And maintaining TERT-related anti-apoptotic function might be one mechanism underlying the protective effect of curcumin on ACR-intoxicated brains.

## Data Availability

The datasets supporting the conclusions of this article are included within the article. The raw data can be requested from the corresponding author.

## Abbreviations

ACR: Acrylamide
GSH: Glutathione
GSH-Px: Glutathione peroxidase
HE: Hematoxylin and eosin
IL-1β: Interleukin-1β
MDA: Malondialdehyde
ROS: Reactive oxygen species
SOD: Superoxide dismutase
TERT: Telomerase reverse transcriptase
TNF-α: Tumor necrosis factor-α
TUNEL: Terminal deoxynucleotidyl transferase mediated dUTP nick end labelling
